# Mowat-Wilson Syndrome: The First Clinical and Molecular Report of an Indonesian Patient

**DOI:** 10.1155/2012/949507

**Published:** 2012-12-01

**Authors:** Farmaditya E. P. Mundhofir, Helger G. Yntema, Ineke van der Burgt, Ben C. J. Hamel, Sultana M. H. Faradz, Bregje W. M. van Bon

**Affiliations:** ^1^Division of Human Genetics, Center for Biomedical Research (CEBIOR), Faculty of Medicine, Diponegoro University GSG, 2nd Floor Jl. Dr. Sutomo 14, Semarang, Indonesia; ^2^Department of Human Genetics, Radboud University Nijmegen Medical Centre, P.O. Box 9101, 6500 HB Nijmegen, The Netherlands

## Abstract

Mowat-Wilson syndrome (OMIM 235730) is a genetic condition characterized by moderate-to-severe intellectual disability, a recognizable facial phenotype, and multiple congenital anomalies. The striking facial phenotype in addition to other features such as severely impaired speech, hypotonia, microcephaly, short stature, seizures, corpus callosum agenesis, congenital heart defects, hypospadias, and Hirschsprung disease are particularly important clues for the initial clinical diagnosis. All molecularly confirmed cases with typical MWS have a heterozygous loss-of-function mutation in the zinc finger E-box protein 2 (*ZEB2*) gene, also called *SIP1* (Smad-interacting protein 1) and *ZFHX1B*, suggesting that haploinsufficiency is the main pathological mechanism. Approximately 80% of mutations are nonsense and frameshift mutations (small insertions or deletions). About half of these mutations are located in exon eight. Here, we report the first Indonesian patient with Mowat-Wilson syndrome confirmed by molecular analysis.

## 1. Introduction

Mowat-Wilson syndrome (MWS; OMIM 235730) is a rare genetic condition described by Mowat et al. in 1998, who reported a series of six children with intellectual disability (ID), striking facial features, and variable multiple congenital anomalies (MCA) [1]. All molecularly confirmed cases with typical MWS have a heterozygous loss of function mutation in the zinc finger E-box protein 2 (*ZEB2*) gene, also called *SIP1* (Smad-interacting protein 1) and *ZFHX1B* [2]. To date, about 200 molecularly proven MWS cases with over 100 different *ZEB2* mutations have been reported [3]. 

The facial features are the most important diagnostic clue for the initial clinical diagnosis and provide a hallmark for *ZEB2* mutation analysis [4]. Establishing a molecular diagnosis is important for the patients and their families as it allows reliable genetic counseling for their families and a better clinical management of the patients. Here, we report the first Indonesian patient with molecularly confirmed MWS. 

## 2. Case Presentation

**Figure 1:**
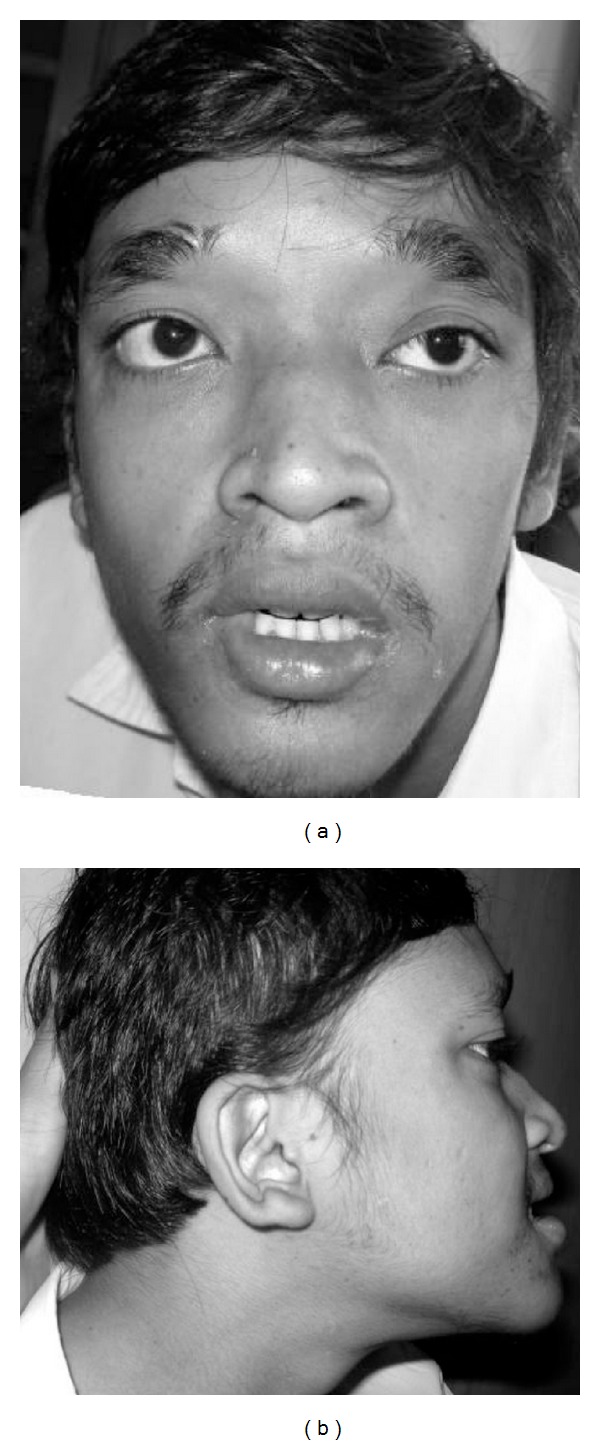
Photograph of Indonesian patient with Mowat-Wilson syndrome. Our patient in his 19 years of age showed striking facial gestalts of MWS such as large eyebrows with medial flaring (a) and uplifted ear lobules (b). Other dysmorphisms such as long face, deep-set eyes, upward slanting palpebral fissures, hypertelorism, strabismus, saddle nose with prominent rounded nasal tip, prominent columella, low-set and posteriorly rotated ears, prominent and triangular pointed chin, small mouth, full lips, and prognathia are noted.

The patient was a nineteen-year-old male with severe ID. He was the third son of nonconsanguineous, healthy, Javanese parents and family history was unremarkable. The patient was born at term after an uneventful pregnancy with a weight of 3200 g (25th centile) and length 50 cm (50th centile). He showed hypotonia and delayed developmental milestones. He started to sit at 20 months of age. At two years of age, he developed recurrent generalized seizures and was commenced on valproic acid, which brought his epilepsy under control. He started to walk at four years of age and spoke his first words at the age of five years. He had recurrent otitis media. Speech consisted of only a few words and he often communicated using sign language. He showed happy behavior with frequent smiling. In addition, he showed repetitive hand movements. On physical examination, his weight was 45 kg (<3rd centile), height 161 cm (<3rd centile), and head circumference 53 cm (<3rd centile). Facial dysmorphisms included a long face, deep-set eyes, large eyebrows with medial flaring, hypertelorism, strabismus, saddle nose with prominent rounded nasal tip, prominent columella, low-set and posteriorly rotated ears, uplifted ear lobules, a prominent narrow pointed chin, a small mouth, and prognathism (Figure 1). In addition, he had tapered and slender fingers, prominent interphalangeal joints, and bilateral pes planus. Generalized hypotonia and hyperreflexia were observed. Heart auscultation was normal. 

**Figure 2:**
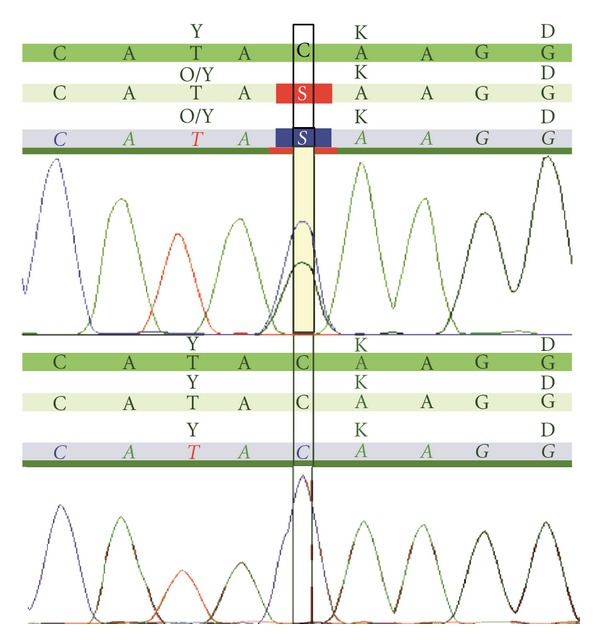
Electropherogram of molecular analysis in the patient sample. The upper panel shows the heterozygous c.1965C>G (p.Tyr652X) mutation and the lower panel shows the wild type (control). The “S” on the electropherogram represents the C/G heterozygote.

The individual was part of a larger series of 527 Indonesian individuals with ID from schools and institutions, whose conventional karyotyping, *FMR1* gene analysis, and subtelomeric MLPA were normal [5]. Based on the clinical features, MWS was suspected. Therefore, molecular analysis of the *ZEB2 *gene was warranted. Sanger sequencing of all coding exons and surrounding splice sites of the *ZEB2 *gene was performed as described below. The genomic DNA reference sequence was NM_014795.2. PCR of exon eight was performed using primers CTTTACTTGGGTTTCCCACC (forward) and GGGGCTTGTCATTCCTT (reverse). One hundred nanograms of DNA solution (1 *μ*L) were added into PCR mixture, which contained 7.6 *μ*L of 360 PCR master mix (Applied Biosystem), 0.5 *μ*L of primers working solution, and 6 *μ*L of H_2_O. Amplification was performed using PCR System 9700 (Applied Biosystem) with the following protocol. PCR was initiated by 10′ denaturation at 95°C, followed by 35 PCR cycles (30^”^ 95°C, 30^”^ 60°C, 60^”^ 72°C) and 7′ final elongation at 72°C. The result was analyzed on ABI 3730 analyzer (Applied Biosystem). Sequence result was compared to published reference sequence (rs148709333) using SEQPilot software version 3.2.1.0 (JSI medical system). In exon eight, a nonsense mutation has been detected, changing a TAC codon (coding for a tyrosine) into a TAG stopcodon; c.1965C>G (p.(Tyr652*)) (nomenclature according to the HGVS guidelines; http://www.hgvs.org/mutnomen/) (Figure 2). To our knowledge, this mutation has not been reported before.

## 3. Discussion

This is the first report of an Indonesian individual with MWS confirmed by molecular genetic testing. Although nonsense mutations account for more than 40% of known *ZEB2* mutations and approximately 50% of these are localized in exon eight [6], the particular mutation detected in the patient described in this paper (c.1965C>G; p.Tyr652*) has not been reported before. 

Most clinical features of our patient, who had severe ID, a distinct facial gestalt, microcephaly, and seizures, are consistent with those described in the literature (Table 1). Brain imaging and echocardiography could not be performed since he is living in the country with minimal health facilities. Symptoms of Hirschsprung disease (HSCR) such as constipation, dysphagia, and poor appetite were not reported in our patient, but the prevalence of these symptoms in other publications ranged from the majority of individuals [1, 2] to 50% of cases [4, 6]. Early diagnosis, intervention, and targeted management are necessary for a better health and life quality of individuals with MWS. However, as this syndrome is rare and recently described, the knowledge of the clinical complications and natural history is still developing [7]. 

In summary, we report the first Indonesian MWS case with a novel *ZEB2* mutation. Our patient showed similar dysmorphism to previously reported cases, although several major associated features were not present such as HSCR, congenital heart defect (CHD), and hypospadia. Despite the availability of molecular diagnostic tests in several parts of the world, the recognition of clinically well-defined syndromes will remain very important in countries with limited diagnostic facilities such as Indonesia. The publication of cases with recognizable facial features is therefore of great importance in order to make local pediatricians aware of rare conditions like Mowat-Wilson syndrome, allowing more clinical diagnoses in the future.

## Figures and Tables

**Table 1:** Clinical features of our patient compared to those in published cases of MWS with proven *ZEB2 *mutations.

Clinical features	Our patient	Mowat-Wilson syndrome*
*ZEB2 *mutations	+	100%
Intellectual disability	+	100%
Typical facial gestalt	+	97%
Microcephaly	+	81%
Seizures	+	73%
HSCR	−**	57%
CHD	−**	52%
Hypospadias	−	52%
Short stature	+	46%
Hypoplasia or agenesis of CCA	NT	43%
Cryptorchidism	−	36%
Constipation	−	26%
Pyloric stenosis	−	4.7%
Eye anomalies	−	4.1%
Cleft palate	−	2.9%

*Adapted from Garavelli and Mainardi (2007) [4].

**Symptoms not observed although the gold standard diagnosis has not been performed.

NT: Not Tested, HSCR: Hirschprung Disease, CHD: Congenital Heart Defect, CCA: Corpus Callosum.
